# Supplementary material for: Multi‐dataset identification of innovative feature genes and molecular mechanisms in keratoconus
Source: J Cell Mol Med. 2024 Sep 19;28(18):e70079. doi: 10.1111/jcmm.70079 (PMC11412914; doi:10.1111/jcmm.70079)
Supplement: Supplementary file 2 — Material S2. [file JCMM-28-e70079-s002.zip › 7.GSEA analysis/1.ARL11/KEGG_AUTOIMMUNE_THYROID_DISEASE.html]

Details for gene set KEGG\_AUTOIMMUNE\_THYROID\_DISEASE[GSEA]

|  || Dataset | ARL11.group.cls#h\_versus\_l |
| Phenotype | group.cls#h\_versus\_l |
| Upregulated in class | l |
| GeneSet | KEGG\_AUTOIMMUNE\_THYROID\_DISEASE |
| Enrichment Score (ES) | -0.44813007 |
| Normalized Enrichment Score (NES) | -1.228147 |
| Nominal p-value | 0.17058824 |
| FDR q-value | 1.0 |
| FWER p-Value | 0.98 |
Table: GSEA Results Summary

  

Fig 1: Enrichment plot: KEGG\_AUTOIMMUNE\_THYROID\_DISEASE      
 Profile of the Running ES Score & Positions of GeneSet Members on the Rank Ordered List

  

| SYMBOL | TITLE | RANK IN GENE LIST | RANK METRIC SCORE | RUNNING ES | CORE ENRICHMENT || 1 | HLA-DRB5 | na | 2778 | 0.251 | -0.0417 | No |
| 2 | GZMB | na | 2928 | 0.244 | -0.0086 | No |
| 3 | IFNA6 | na | 3473 | 0.220 | 0.0094 | No |
| 4 | HLA-DOA | na | 3812 | 0.207 | 0.0314 | No |
| 5 | IFNA14 | na | 4269 | 0.191 | 0.0475 | No |
| 6 | IFNA16 | na | 4914 | 0.169 | 0.0547 | No |
| 7 | IFNA4 | na | 6226 | 0.131 | 0.0369 | No |
| 8 | TG | na | 6853 | 0.115 | 0.0365 | No |
| 9 | HLA-DRB4 | na | 7218 | 0.107 | 0.0424 | No |
| 10 | HLA-DRA | na | 7656 | 0.099 | 0.0449 | No |
| 11 | HLA-DPA1 | na | 7767 | 0.097 | 0.0566 | No |
| 12 | HLA-DRB1 | na | 7965 | 0.093 | 0.0651 | No |
| 13 | CD86 | na | 8057 | 0.091 | 0.0765 | No |
| 14 | HLA-DQB1 | na | 8352 | 0.086 | 0.0811 | No |
| 15 | TSHR | na | 8791 | 0.078 | 0.0804 | No |
| 16 | HLA-F | na | 10625 | 0.052 | 0.0356 | No |
| 17 | HLA-G | na | 14146 | 0.018 | -0.0632 | No |
| 18 | HLA-DPB1 | na | 14439 | 0.016 | -0.0693 | No |
| 19 | HLA-E | na | 14680 | 0.014 | -0.0741 | No |
| 20 | IFNA2 | na | 15087 | 0.011 | -0.0842 | No |
| 21 | CTLA4 | na | 15578 | 0.007 | -0.0973 | No |
| 22 | HLA-C | na | 15719 | 0.006 | -0.1004 | No |
| 23 | FASLG | na | 15958 | 0.004 | -0.1067 | No |
| 24 | HLA-DMA | na | 17342 | -0.006 | -0.1457 | No |
| 25 | HLA-DOB | na | 17625 | -0.008 | -0.1527 | No |
| 26 | HLA-A | na | 17972 | -0.010 | -0.1611 | No |
| 27 | HLA-B | na | 19514 | -0.021 | -0.2024 | No |
| 28 | CGA | na | 19555 | -0.021 | -0.2004 | No |
| 29 | CD28 | na | 19606 | -0.021 | -0.1986 | No |
| 30 | HLA-DRB3 | na | 19647 | -0.021 | -0.1964 | No |
| 31 | TSHB | na | 22751 | -0.047 | -0.2786 | No |
| 32 | TPO | na | 23798 | -0.060 | -0.2997 | No |
| 33 | HLA-DQA2 | na | 24037 | -0.063 | -0.2969 | No |
| 34 | FAS | na | 24099 | -0.064 | -0.2889 | No |
| 35 | CD40 | na | 26388 | -0.096 | -0.3401 | No |
| 36 | HLA-DMB | na | 27501 | -0.119 | -0.3540 | No |
| 37 | IL2 | na | 30766 | -0.213 | -0.4155 | Yes |
| 38 | HLA-DQA1 | na | 30949 | -0.219 | -0.3873 | Yes |
| 39 | CD80 | na | 31382 | -0.235 | -0.3638 | Yes |
| 40 | IFNA8 | na | 31555 | -0.242 | -0.3318 | Yes |
| 41 | CD40LG | na | 31605 | -0.244 | -0.2958 | Yes |
| 42 | IL5 | na | 32563 | -0.292 | -0.2787 | Yes |
| 43 | IL10 | na | 32834 | -0.310 | -0.2391 | Yes |
| 44 | IFNA10 | na | 33230 | -0.339 | -0.1985 | Yes |
| 45 | IFNA7 | na | 33254 | -0.341 | -0.1470 | Yes |
| 46 | IL4 | na | 33709 | -0.380 | -0.1019 | Yes |
| 47 | PRF1 | na | 33908 | -0.407 | -0.0454 | Yes |
| 48 | IFNA21 | na | 34187 | -0.451 | 0.0156 | Yes |
Table: GSEA details [plain text format]

  

Fig 2: KEGG\_AUTOIMMUNE\_THYROID\_DISEASE      
 Blue-Pink O' Gram in the Space of the Analyzed GeneSet

  

Fig 3: KEGG\_AUTOIMMUNE\_THYROID\_DISEASE: Random ES distribution      
 Gene set null distribution of ES for **KEGG\_AUTOIMMUNE\_THYROID\_DISEASE**
